# Insight into Alternative Approaches for Control of Avian Influenza in Poultry, with Emphasis on Highly Pathogenic H5N1

**DOI:** 10.3390/v4113179

**Published:** 2012-11-19

**Authors:** E. M. Abdelwhab, Hafez M. Hafez

**Affiliations:** Institute of Poultry Diseases, Free Berlin University, Königsweg 63, 14163 Berlin, Germany; Email: hafez@vetmed.fu-berlin.de

**Keywords:** influenza, H5N1, control

## Abstract

Highly pathogenic avian influenza virus (HPAIV) of subtype H5N1 causes a devastating disease in poultry but when it accidentally infects humans it can cause death. Therefore, decrease the incidence of H5N1 in humans needs to focus on prevention and control of poultry infections. Conventional control strategies in poultry based on surveillance, stamping out, movement restriction and enforcement of biosecurity measures did not prevent the virus spreading, particularly in developing countries. Several challenges limit efficiency of the vaccines to prevent outbreaks of HPAIV H5N1 in endemic countries. Alternative and complementary approaches to reduce the current burden of H5N1 epidemics in poultry should be encouraged. The use of antiviral chemotherapy and natural compounds, avian-cytokines, RNA interference, genetic breeding and/or development of transgenic poultry warrant further evaluation as integrated intervention strategies for control of HPAIV H5N1 in poultry.

## Abbreviations

AIV: avian influenza virus
ChIFN-α : chicken interferon alpha
ChIL : chicken interleukin
ECE: embryonated chicken eggs
HA : hemagglutinin
HPAIV : highly pathogenic avian influenza virus
IFN : interferon
LPAIV : low pathogenic avian influenza virus
Mx : myxovirus
NA : neuraminidase
NAIs : neuraminidase inhibitors
rFPV : recombinant fowl pox virus
RIG-I : retinoic acid-inducible gene I
RNA : ribonucleic acid
RNAi : RNA interference
siRNA : short-interfering RNA
SPF : specific pathogen free
TLR : Toll-like receptors

## 1. Introduction

Influenza A virus, the only *orthomyxovirus* known to infect birds, are negative-sense, single-stranded, enveloped viruses contain genomes composed of eight separate ribonucleic acid (RNA) segments encode for at least 11 viral proteins. Two surface glycoproteins; hemagglutinin (HA) and neuraminidase (NA) are playing a vital role in attachment and release of the virus, respectively [1]. The 17 HA and 10 NA subtypes of avian influenza viruses (AIV) are classified according to their pathogenicity for poultry into low pathogenic AIV (LPAIV) result in mild or asymptomatic infections and highly pathogenic AIV (HPAIV) causing up to 100% morbidity and mortality [2,3]. To date, some strains of H5 or H7 subtypes fulfilled the defined criteria of high pathogenicity which potentially evolve from low virulent precursors [4]. Constant genetic and antigenic variation of AIV is an intriguing feature for continuous evolution of the virus in nature [5]. Gradual antigenic changes due to acquisition of point mutations known as “antigenic drift” are commonly regarded to be the driving mechanism for influenza virus epidemics from one year to the next. However, possible “antigenic shift or reassortment” of influenza virus occurs by exchange genes from different subtypes is relatively infrequent, however it results in severe pandemics [6].

HPAIV H5N1 is responsible for magnificent economic losses in poultry industry and poses a serious threat to public health [7,8]. Measures to control the virus in domestic poultry are the first step to decrease risks of human infections [9,10]. Enhanced biosecurity measures, surveillance, stamping out and movement restriction as basic principles for control of HPAIV H5N1 epidemics in poultry [11] has not prevented the spread of the virus since 1997 [12,13]. Recently, vaccines have been introduced in some developing countries as a major control tool to reduce the overwhelming socioeconomic impact of HPAI H5N1 outbreaks in poultry [13]. Different types of inactivated vaccines and to lesser extent recombinant live virus vaccines are already in use that decrease shedding of the virus, morbidity, mortality, transmissibility, increase resistance to infection, lower virus replication and limit decrease in egg production [2,14]. 

Nevertheless, several challenges facing the efficiency of the vaccine to control the HPAIV H5N1 outbreaks have been reported: (1) Vaccine is HA subtype specific and in some regions where multiple subtypes are co-circulating (*i.e.*, H5, H7 and H9), vaccination against multiple HA subtypes is required [15]. (2) Vaccine-induced antibodies hinder routine serological surveillance and differentiation of infected birds from vaccinated ones requires more advanced diagnostic strategies [16]. (3) Vaccination may prevent the clinical disease but can’t prevent the infection of vaccinated birds, thus continuous “silent” circulation of the virus in vaccinated birds poses a potential risk of virus spread among poultry flocks and spillover to humans [17,18,19]. (4) Immune pressure induced by vaccination on the circulating virus increases the evolution rate of the virus and accelerates the viral antigenic drift to evade the host‑immune response [20,21,22,23,24]. (5) After emergence of antigenic variants, the vaccine becomes useless and/or inefficient to protect the birds and periodical update of the vaccine is required [20,25,26,27,28]. (6) Vaccine-induced immunity usually peaks three to four weeks after vaccination and duration of protection following immunization remains to be elucidated [29]. (7) Maternally acquired immunity induced by vaccination of breeder flocks could interfere with vaccination of young birds [30,31,32,33,34]. (8) Other domestic poultry (*i.e.*, ducks, geese, turkeys), zoo and/or exotic birds even within the same species (*i.e.*, Muscovy *vs.* Pekin ducks) respond differently to vaccination which have not yet been fully investigated compared to chickens [35,36,37,38,39,40,41,42]. (9) Concomitant or prior infection with immunosuppressive pathogens or ingestion of mycotoxins can inhibit the immune response of AIV‑vaccinated birds [43,44,45,46]. (10) And last but not least, factors related to vaccine manufacturing, quality, identity of vaccine strain, improper handling and/or administration can be decisive for efficiency of any AIV vaccine [2,29]. 

Therefore, presence of new alternative and complementary strategies target different AIV serotypes/subtypes/drift-variants should be encouraged. This review aims to give an insight into possible alternative approaches for control of AIV in poultry particularly against the HPAI H5N1 subtype.

## 2. Antivirals

### 2.1. Chemotherapy

The use of chemotherapeutic agents for control of AIV in poultry was concurrently studied just after discovering their anti-microbial effects [47,48]. However, during the last three decades more attention was paid to the commonly used antivirals, M2 blocker and neuraminidase inhibitors (NAIs), in control of human influenza viruses to be used in eradication of AIV in poultry. 

#### 2.1.1. M2 Blockers (Adamantanes)

Amantadine hydrochloride and rimantadine are two M2 blockers which interrupt virus life cycle by blocking the influx of hydrogen ions through the M2 ion-channel protein and prevent uncoating of the virus in infected host-cells [49,50,51]. The prophylactic activity of **amantadine** in poultry was firstly studied by Lang *et al.* [52] in experimentally infected turkeys with an HPAIV H5N9 isolated in 1966 from Ontario, Canada. Optimum prophylaxis was obtained only when amantadine was administered in an adequate, uninterrupted and sustained amount from at least 2 days pre-infection to 23 days post‑infection. During H5N2 outbreaks in Pennsylvania, USA in early 1980s, one of control proposals was the use of amantadine as a therapeutic and/or prophylactic approach. Under experimental condition, amantadine given in drinking water was efficacious to decrease morbidity, mortality, transmissibility and limit decrease in egg production [53,54]. Nonetheless, all recovered birds were susceptible to reinfection [52,54,55,56] and subclinical infection was reported in most of treated birds [52]. Importantly, amantadine lost its effectiveness as amantadine-resistant mutants emerged within 2–3 days of treatment and killed all in-contact chickens. Amantadine-resistant strains were irreversible, stable and transmissible with pathogenic potential comparable to the wild-type virus. Even more, the resistant mutants replaced the wild-type virus and became dominant [55,56,57]. It is worth pointing out that several subtypes of AIV including the HPAIV H5N1 that currently circulate in both humans and birds around the world are mostly resistant to amantadine [58,59,60,61,62,63,64,65]. Since the late 1990s, positive selection of amantadine-resistant HPAI H5N1 viruses in poultry in China has been proven to be increased due to extensive illegal application of the relatively inexpensive amantadine by some farmers to control HPAIV H5N1 (and LPAIV H9N2) infections in chickens [62,66,67,68,69]. Hence, rapid selection of amantadine-resistant variants threatens the effective use of the drug for control of human influenza epidemics and/or pandemics [70], therefore the extra-label use of amantadine in poultry was banned by all concerned international organizations [71,72]. The second M2 blocker is **rimantadine**. Because of the unavailability of rimantadine in most countries, its use in poultry is not reported until now in the field. However, Webster *et al.* [73] mentioned that rimantadine administered in drinking water was efficacious against HPAIV H5N2 infection in experimentally infected chickens. Nonetheless, the emergence of rimantadine-resistant variants was comparable to amantadine.

#### 2.1.2. Neuraminidase Inhibitors (NAIs)

So far, there are two main NAIs, oseltamivir (Tamiflu®) and zanamivir (Relenza®) have been licensed for influenza treatment in human in several countries [74]. When exposed to NAIs, influenza virions aggregate on the host cell surface preventing their release and allow the host immune system to eliminate the virus [75,76]. In the early 2000s, **oseltamivir** was discovered as a potent and selective inhibitor of the NA enzyme of influenza viruses [50]. It is currently the drug of choice for the treatment of influenza virus infections in human and being stockpiled in many countries in anticipation of a pandemic [77]. Generally, AIV including H5N1 are sensitive to oseltamivir [78] and a small number of H5N1 strains isolated from avian and human origin have been reported to exhibit resistance to oseltamivir [79,80,81,82,83,84]. Oral application of oseltamivir via drinking water reduced the morbidity, mortality, virus excretion and chicken-to-chicken transmission in HPAIV H5N2 experimentally infected chickens [85]. Oseltamivir was non-toxic for chicken embryos and prevented the replication of an HPAIV H7N1 in inoculated eggs [86]. An effective prophylactic administration of oseltamivir in experimentally infected chickens and ducks with LPAI H9N2 and H6N2 viruses was also reported [87]. Although it is very plausible that oseltamivir-resistance mutants emerge after application in poultry, however none of the few studies conducted to evaluate efficacy of oseltamivir in avian species reported emergence of resistant strains. In nature, oseltamivir-resistant H5N1 viruses isolated from domestic and wild birds emerged probably due to spontaneous mutations rather than exposure to oseltamivir [80,88,89,90]. Administration of oseltamivir during an outbreak in commercial flocks is extremely expensive but it could be useful to protect valuable birds [86,87]. On the other hand, **zanamivir** is currently approved in 19 countries for the treatment and prophylaxis of human influenza [50]. Although, development of zanamivir-resistance in poultry is rare [91], it is not effective in preventing a severe outcome and chicken-to-chicken transmission of an HPAIV H5N2 in experimental chickens [85]. 

### 2.2. Natural Antivirals

#### 2.2.1. Herbs

Unlimited herbs products contain polyphenols, flavonoids, alkaloids or lignans, mostly from traditional Chinese medicine, offer promise as adjuncts or alternatives to the current anti-influenza chemotherapy [92,93]. Generally, complementary medicine for treating or preventing influenza or influenza-like illness in human seems to be cultural practice differs from nation to nation [94,95,96]. Innumerable herbs species with potential inhibitory effects on replication of influenza viruses using *in‑vitro* cell culture methods and embryonated eggs or *in-vivo* mouse models were frequently described [97,98,99,100,101,102,103,104,105,106,107,108,109,110,111,112,113,114,115,116,117,118,119,120,121,122,123]. 

In poultry, antiviral and immunoadjuvant effects of several plants and/or its derivatives have been investigated. In addition to its antiviral activity, these extracts often have anti-bacterial, anti-fungal, anti-inflammatory, anti-oxidant and/or analgesic properties which may provide alternative natural broad-spectrum therapy for control of AIV in poultry farms [124,125,126,127]. Sood *et al.* [127] found that *Eugenia jambolana* extracts had 100% virucidal activity against HPAIV H5N1 in tissue culture and *in‑ovo* inoculated chicken embryonated eggs (ECE). Menthol, eucalyptol and ormosinine probably have inhibitory effect on H5 viruses due to strong interactions ability with the viral HA protein [128]. NAS preparation, a Chinese herbal medicine, prevented H9N2 virus-induced clinical signs in treated chickens; however transmission of the virus to untreated chickens was not interrupted [129]. Likewise, eucalyptus and peppermint essential oils preparations protected broilers against H9N2 virus infections [130,131]. Moreover, application of lyophilized green tea by-product extracts namely catechins in feed or drinking water reduced H9N2 virus replication and excretion in experimentally infected chickens in a dose-dependent manner [132]. In addition, green tea extract was comparable to amantadine in protection of chicken embryos against H7N3 subtype [120]. Catechins alter the infectivity of influenza viruses probably not only by direct interaction with viral HA but also by inhibition of viral RNA synthesis in cell culture [133]. Furthermore, Liu *et al.* [134] found that statin/caffeine combination was as effective as oseltamivir in reduction HPAIV H5N1-induced lung damage and viral replication in mice.

The immunoadjuvant effect of some herbal extracts as feed additives on the humoral immune response induced by inactivated AIV vaccination in poultry has been studied. Oral administration of *ginseng* stem-and-leaf saponins in drinking water or *Hypericum perforatum L.* as a dietary supplement significantly enhanced serum antibody response to inactivated H5N1 or H9N2 vaccines in chickens [135,136,137]. The *Cochinchina momordica* seed extract, Chinese medicine plant, when combined with an inactivated H5N1 vaccine as adjuvant increased significantly the immune response and daily weight gain of two weeks old chickens [138]. On the contrary, herbal extracts of *Radix astragali*, *Radix codonopis*, *Herba epimedii* and *Radix glycyrrizae* in drinking water did not improve chicken immune response to H5-AIV vaccination [139], likewise diet supplementation with fresh garlic powder had no effect on the humoral immune response of chickens vaccinated with an inactivated H9N2 vaccine [140]. 

Yet, some derivatives (*i.e.*, ginseng saponins) require four to six years to harvest and is very expensive on the market [135]. Methods of the extraction and preparation of the crude extracts and its purity greatly influence the inhibition activity of some herbs against AIV [132,133]. Moreover, batch‑to-batch variations due to variable growth conditions at the plantations have been considered a limiting factor for treatment of influenza [124]. Evident that mutation in the H5 gene probably affects inhibitor binding of some herbs was reported [128]. In addition, *in-vitro* experiments and animal models to confirm the direct antiviral activities against influenza virus are limited [141]. Moreover, comprehensive investigations of herb-drug interactions, potential toxicity, heterogeneity of herbs species, plant parts (*i.e.*, aerial *vs.* root) and biochemical data identifying the active components are inadequately described [142]. 

#### 2.2.2. Probiotics

A number of studies have reported the efficacy of probiotic lactic acid bacteria such as *Streptococcus thermophiles*, several *Lactobacillus* and *Bifidobacterium* species to enhance the immune response and to protect mice against different influenza strains/serotypes [143,144,145,146,147,148,149,150,151,152]. Although probiotics are widely used in poultry to improve innate and adaptive immunity [153,154,155], there is a paucity of information on its ability to ameliorate AIV infections. *Lactobacillus plantarum* KFCC11389P was as effective as oseltamivir to neutralize the H9N2 virus in ECE and slightly reduced amount of tracheal virus excretion in oral-fed experimentally infected chickens [156]. Out of 220 screened bacterial strains, Seo *et al.* [157] found that *Leuconostoc mesenteroides *YML003 had highly anti-H9N2 activity in cell culture and ECE. Decrease cloacal excretion of the virus and a significant increase in the cytokine IFN-gamma in experimentally infected chickens were observed. Ghafoor and co-workers [158] showed that multi-strains commercial probiotic protexin® (various *Lactobacillus* sp., *Enterococcus faecium*, *Bifidobacterium bifidum*, *Candida pintolepesii *and *Aspergillus oryzae*) improved immune response of broiler chickens to H9N2 vaccination and prevented the mortality and morbidity. On the other hand, dual use of *Lactobacillus spp.* or *Lactococcus lactis* as a vector for vaccine production and immunomodulation bacteria has been successfully constructed and protected mice against HPAIV H5N1 [159,160], such experiments should be evaluated in poultry.

## 3. Molecular Approaches for Control of AIV

### 3.1. Avian-Cytokines

Chicken cytokines such as chicken interferon-alpha (ChIFN-α), chicken interleukins (ChIL) and Toll-like receptors (TLR) are essential components of chicken’s innate immune system which play a vital role against virus infections [15,161,162,163]. An innovative application of ChIFN-α to antagonize AIV infection in poultry through direct oral feeding or drinking water has received more attention than other components [164,165,166,167,168]. Sekellick *et al.* [169] showed that up to 60% of investigated AIV population belonged to the HPAI H5N9 subtype were highly sensitive to the inhibitory effects of ChIFN-α. Interestingly, both IFN-sensitive and -resistant clones were obtained after passage of the resistant clones in the presence of IFN which indicated that resistance to ChIFN-α was transient and did not result from stable genetic changes. Xia *et al.* [170] cloned the ChIFN-α gene from three different chicken lines and studied their efficacy against H9N2 viruses *in-ovo *and *in-vivo*. Up to 70% of *in-ovo* treated chicken embryos were protected against H9N2 virus infection in dose dependent manner. Moreover, chickens received ChIFN-α by oculonasal inoculation at one day of age were protected from death upon H9N2 virus infection given 24 hours later. Findings of Meng and co‑workers [166] showed that oral administration of exogenous ChIFN-α was effective to prevent and treat chickens experimentally infected with an H9N2 virus. It potentially reduced the viral load in trachea and resulted in rapid recovery of the body weight gain. In another study, White Leghorn (WL) chickens received ChIFN-α in drinking water for 14 successive days augmented detectable humoral anti-influenza antibodies after exposure to a low dose of an LPAIV H7N2 infection [164]. Thus, it has been suggested that regular water administration of ChIFN-α can create “super-sentinel” chickens to detect early infections with few amount of LPAIV [164]. 

Furthermore, oral administration of live attenuated *Salmonella enterica* serovar Typhimurium expressing ChIFN-α alone or in combination with ChIL-18 significantly reduced clinical signs induced by H9N2 virus and decreased the amount of virus load in cloacal swabs and internal organs [171,172]. Likewise, chicken immunized with a recombinant fowl pox virus (rFPV) vaccine expressing both the HA gene of H9N2 virus and ChIL-18 survived challenge with an H9N2 virus and did not excrete any virus in swab samples and/or internal organs in comparison to non-vaccinated birds [173]. Also, rFPV expressing the H5, H7 and ChIL-18 genes produced significantly higher humoral and cellular mediated immune response and protected specific pathogen free chickens (SPF) and WL chickens against challenge with an HPAIV H5N1. Vaccinated birds had no virus shedding and showed significant increase in body weight gain [174]. So far, efficiency of avian-cytokines to limit AIV infection has not been adequately studied in other avian species. The duck IL-18 and IL-2 genes had been identified and shown to have 85% and 55% nucleotide identity to the chicken equivalents, respectively. Intramuscular inoculations of the duck IL-18 or IL-2 enhanced the humoral immune response of ducks vaccinated with H5N1 or H9N2 inactivated vaccines, respectively [175,176]. Likewise, the recombinant goose IL-2 strengthens goose humoral immune responses after vaccination using H9N2 inactivated vaccine [177].

The TLR-3, TLR-7 and TLR9 are other promising chicken cytokines derivatives that showed broad-spectrum anti-influenza virus activity *in-vitro* and *in-ovo* [178,179,180,181]. Nevertheless, the cost of mass production of chicken cytokines is still too high to be applied in large-scale in poultry industry [165]. Moreover, protein stability, host-specificity and labor associated with mass administration of chicken cytokines under field conditions require significant improvement [172].

### 3.2. RNA Interference (RNAi)

RNAi is a natural phenomenon used by many organisms as a defense mechanism against foreign microbial invasion, including viruses, that able to wreak potential genetic havoc of the susceptible host [182]. Short-interfering RNA (siRNA) is approximately 21–25 nucleotides specific for highly conserved regions of AIV genomes. It effectively mediates the catalytic degradation of complementary viral mRNAs and results in inhibition of a broad spectrum of influenza viruses replication in cell lines, chicken embryos and mice just before or after initiation of an infection [183,184,185,186,187]. Tompkins and colleagues [188] found that siRNA specific for the NP or PA genes induced full protection of mice against lethal challenge with the HPAI H5N1 and H7N7 subtypes and markedly decreased virus titers in lungs. Likewise, prophylactic use of PA-specific siRNA molecule significantly reduced lung H5N1 virus titers and lethality in infected mice [189]. Moreover, siRNA targeting M2 or NP genes inhibited replication of H5N1 and H9N2 viruses in canine cell line and partially protected mice against HPAV H5N1 [190].

In poultry, Li and others [191] showed that the siRNA targeting NP and/or PA genes inhibited protein expression, RNA transcription and multiplication of HPAIV H5N1 in chicken embryo fibroblasts and ECE as well as prevented apoptosis of infected cells. Likewise, chicken cell line transfected with RNAi molecules specific for the NP or PA of AIV showed decrease the levels of NP mRNA and infective titre of an H10N8 quail virus [192]. Also, NP-specific siRNA reduced H5N1 virus replication in cell culture and ECE [186]. Moreover, siRNA molecules targeting the NP, PA and PB1 genes interfered with replication of H1N1 virus in ECE [184]. 

In contrast to AIV vaccines, siRNA might not require an intact immune system [193] which is very important particularly in developing countries where a number of immunosuppressive agents are endemic in poultry. In addition, siRNA molecules targeting the highly conserved regions in influenza genome potentially remain effective regardless AIV subtype/serotype variations and despite antigenic drift and shift of AIV [193,194]. Moreover, it has also the potential to reduce the emergence of viable resistant variants [10], in this regard combinations of siRNA molecules “cocktail” targeting several genes/regions may be used simultaneously [195,196]. Furthermore, there is no risk of recombination between siRNA nucleotides and circulating influenza viruses, hence siRNA is complementary to the influenza virus genome [10]. Moreover, the siRNA dose required for inhibition of AIV is very low (sub-nanomoles) [195]. Nevertheless, arise of mutants with the ability to evade the inhibition effect of siRNA are not fully excluded [193]. Unfortunately, there is no stretch of conserved nucleotides in the NA and HA genes sufficient to generate specific siRNA due to extensive variations in these genes among AIV from different species [195]. The siRNA molecules are quickly degraded *in-vivo* affording a transient short-term protection and multiple-dose is required [192]. None of the siRNAs must share any sequence identity with the host genome to avoid non-specific RNAi-induced gene silencing of the host cells [195,197,198,199]. Delivery vehicle of siRNA to the site of infection is a major constraint [200,201] remained to be investigated on flock-level in poultry. There is accumulating evidence that siRNA is efficient to inhibit influenza virus replication *in-vitro*, however *in-vivo* studies still missing. Research studies focus on mass application of siRNA in poultry as a spray or via drinking water are highly recommended [202]. 

### 3.3. Host Genetic Selection

The host genetics play a pivotal role in susceptibility to influenza including the HPAIV H5N1 which is frequently studied in mice models as reviewed by Horby *et al.* [203]. Indeed, the impact of host genetic selection on resistance to AIV infections in poultry has not yet been fully determined. The on-going H5N1 virus epidemics have raised concerns in respect to influenza-resistant chickens either by selective breeding or genetic modification.

#### 3.3.1. Natural Resistance

It has been supposed that fast-growing domestic birds have reduced immune competence against several viral diseases and resistant breeds are mostly poor producers [204]. Natural resistance or less susceptibility of some species/breeds of birds to AIV is not uncommon. In an experiment, five chicken lines were infected with an HPAIV H7N1. Three lines showed high susceptibility to the virus while two lines showed some resistance and survived the infection [205]. Swayne *et al.* [206] observed that an LPAIV H4N8 produced more severe lesions in commercial and SPF WL chickens than in 5 week‑old commercial broiler chickens suggesting that SPF WL chickens are more susceptible than broilers to this strain. Thomas *et al.* [207] suggested that WL chickens may be more susceptible to an H3N2 virus of swine origin than White Plymouth Rock broiler-type chickens. On the contrary, severe lesions in commercial broiler chickens compared to SPF was observed after experimental infection with a Jordanian H9N2 isolate [208]. Some wild duck species, particularly mallards, are more resistance to HPAIV H5N1 than others [209]. Conversely, dabbling ducks and white fronted goose were more frequently infected with AIV than other wild ducks and geese, respectively [210]. Wood ducks were the only species to exhibit illness or death between different species of experimentally infected wild ducks in a study conducted by Brown and others [211]. 

##### 3.3.1.1. Myxovirus (Mx) Resistance Gene

Myxovirus resistance gene is an interferon-stimulated gene encodes Mx1 protein that able to interfere with AIV replication by inhibiting viral polymerases in the nucleus and by binding viral components in the cytoplasm. The role of the Mx gene in resistance against influenza viruses including the HPAIV H5N1 in mammals is well defined [212,213,214,215,216,217,218]. However, the contribution of avian Mx proteins as antiviral elements in AIV infection in birds is contradictory and worth further exploration. Although intra- and inter-breed/-species Mx variations have been frequently reported [205,219,220,221,222,223,224,225,226], however commercial chicken lines have lower frequencies of the resistant allele compared to the indigenous chicken breeds [219,220,227] probably due to intensive modern breeding techniques [228]. Duck Mx was the first avian Mx protein to be characterized but no antiviral activity against an HPAIV H7N7 when transfected in chicken and mouse cells was obtained [229]. On the contrary, chickens have a single Mx1 gene [230] with multiple alleles [220] encoding a deduced protein with 705 amino acids in length. Notably, results of anti-influenza activity of the Mx1 protein in chickens are contradictory likely due to using variable experimental setups and different AIV strains. Also, a similar disparity has been noted between *in-vitro* and *in-vivo* experiments [205,231]. 

Phenotypic variation in the antiviral activity of Mx gene has been linked to a single amino acid substitution of asparagine (Asn) at position 631 in resistant breeds or serine (Ser) in sensitive ones [219]. The 631Asn identified mostly in Japanese native chicken breeds screened by Ko *et al.* [219] was associated with enhanced antiviral activity to H5N1 virus in transfected mouse fibroblast 3T3 cells. Conversely, results obtained by Benfield *et al.* [232,233] and Schusser *et al.* [234] indicated that neither the 631Asn nor the 631Ser genotypes of chicken Mx1 was able to confer protection against several LPAIV and HPAIV including H5N1 subtype in chicken embryo fibroblasts or ECE. Similarly, Mx1 631Asn had no effect on viral replication after *in-vitro* infection of chicken embryo kidney cells with an LPAIV H5N9 [231]. Moreover, transfected chicken cells expressing chicken Mx protein did not induce resistance to HPAIV H7N7 [235]. *In-vivo*, following intranasal infection with an HPAIV H5N2, chickens carry Asn631 allele showed delayed mortality, milder morbidity and lesser virus excretion than 631Ser homozygotes [231]. Conversely, no correlation was observed between Mx-631 genotypes and susceptibility of chickens to an HPAIV H7N1 as indicated by clinical status and time course of infection [205]. Although, one out of six chicken lines infected with an HPAIV H7N1 had lower mortality, the Mx gene was not involved in this variations among tested chicken lines [236]. Additionally, chickens carry the homozygous Mx resistant allele genotype augmented the lowest HI titer after vaccination with an inactivated H5N2 vaccine compared with chickens that carry the sensitive allele [237].

Taken together, resistance or susceptibility to a disease is usually multifactorial in nature and greatly influenced by both the host and the virus, therefore the role of Mx1 gene merits more in-depth investigation [224,234]. *In-vivo* comparative studies using several native breeds from different countries are required to elucidate the role of Mx1 gene in AIV resistance [231]. 

##### 3.3.1.2. Other Candidate Genes

Apart from the Mx1 gene, resistance or less susceptibility of ducks to AIV infections compared with chickens has been linked to an influenza virus sensor known as retinoic acid-inducible gene I “RIG-I” (a cytoplasmic RNA sensor contribute to AIV detection and IFN production) which is absent in chickens [238,239,240]. This RIG-I gene as a natural AIV resistance gene in ducks could be a promising candidate for creation of transgenic chickens [238]. Moreover, different genes and cytokines have been expressed after infection of chicken and duck cells with several AIV subtypes including HPAIV H5N1 [241,242,243,244]. Additional genetic candidates that contribute to inhibition of AIV replication could be useful in creation of genetically modified chickens such as cyclophilin A [245], ISG15 [246], viperin [247], heat shock cognate protein 70 (Hsc70) [248] or Ebp1 and/or ErbB3-binding protein [249].

#### 3.3.2. Transgenic Chickens

Current advance in molecular biology and genetic manipulation can facilitate the development of influenza-resistant poultry. Increase resistance of cell lines to influenza virus infection using RNA interfering (RNAi) molecules expressed by a lentiviral vector is more efficient transgenic tool than direct DNA injection or oncoretroviral vectors infection [10,250,251]. Recently, creation of AIV built‑in resistant chickens by genetic modification has been experimentally proven by Lyall and colleagues [252]. Chickens equipped with a short-hairpin RNA targets the AIV polymerase binding sites have been created and infected with HPAIV H5N1. Although all infected transgenic birds succumbed to the infection however the virus did not spread to the in-contact transgenic and non‑transgenic cagemates [252]. Applicability in food production, safety regulations and consumer’s preferences are important challenges face development of genetically modified chickens [252,253]. Moreover, AIV is a “master of mutability” and global production of the resistant chickens must be equipped with many decoys target different genes to avoid rapid generation of AIV resistance. In addition, replacement of the commercial flocks with the newly flu-resistant birds is expected to occur within short period due to globalization of the poultry industry however replacement of backyard birds seems to be more complicated [253]. 

## 4. Summary and Perspectives

Epidemics of avian influenza in poultry are a real challenge for the scientific community [12]. Recently, several approaches to control the disease were developed and have yielded promising results. Although beneficial, these approaches face different limitations and restrictions (Table 1). The use of antiviral drugs in poultry could be an ancillary tool to control AIV infections in valuable birds but not in commercial sectors. Fears of kicking out our leading antiviral drugs in control of AIV are increased by adoption of amantadine (and probably oseltamivir) in poultry and transmission of resistant variants to human. On the other hand, limited supply and high costs of oseltamivir preclude its widespread use for poultry. Compliance with other medications, adverse effects and drug residues in eggs, meat and surrounding environment should be investigated. On the other hand, effectiveness of herbal and cytokines-based medications to protect against HPAIV H5N1 should be seriously considered and further investigation *in-vivo* is inevitable.

Molecular approaches including RNAi and transgenic chickens for control of AIV are encouraging. The use of short interfering RNA prevents the replication of AIV seems to be a promising approach; however specificity to the viral genome without interference with the host genome and non‑specifically inhibition of cellular gene activity is critical. Delivery to the host, production costs, mass production and application, storage and handling of the final products are important aspects that remain unresolved. Possibility for arise of mutants with the ability to evade the siRNA activity should also be considered. Genetic resistance to AIV determined by only one point mutation in the Mx gene or complex and multigenic host components as recently determined in mice [254] should be firstly confirmed and secondly elucidation of its relation to the productivity of birds and other diseases must be considered. 

Although a proof-of-principle to produce transgenic chickens has been recently reported, technical, logistic and social constraints are facing development of chicken resistant to AIV. Stable transmission and expression of the transgene from generation to generation require extensive studies. Regulatory approval, mass production, costs and marketing of commercial AIV resistant pedigree lines, consumer preferences and food safety issues need to be carefully and fully addressed. Overall, mutation of the virus in the face of any control approach remains the real challenge. Influenza epidemics and pandemics will likely continue to cause havoc in poultry and human populations, therefore innovative alternative or complementary intervention strategies need to be developed. The ultimate goal of all control (including alternate) strategies must be the eradication of avian influenza. In this context, alternate approaches might be an aid but should not jeopardize surveillance and current control measures.

**Table 1:** Advantages and limitations of different alternative approaches for control of avian influenza viruses in poultry.

Approach		Advantages	Limitations
**Antivirals**	**Chemotherapy**		
	M2 Blockers (Amantadine and Rimantadine) and Neuraminidase inhibitors (Oseltamivir and Zanamivir)	Rapid protection Mass administration (feed, water) Cost-effective for individual birds (amantadine HCL) Suitable for all types of birds against all types of AIV	Hazards of kicking out cornerstone antivirals in case of pandemic Emergence of resistant mutants Require long application period to be effective Expensive in flock level (Oseltamivir) Residues in meat and eggs was not fully addressed Compliance with other medical agents need to be considered
	**Natural Antivirals**		
	Herbs	Direct antiviral activity Immunoadjuvant effect Additional effects as antioxidants, anti-inflammatory, *etc.* No adverse effects on body weight, egg production	Extraction is very expensive Affection with antigenic changes, herb-drug interactions, cytotoxicity and biochemical traits were not fully investigated Extraction methods, preparation, purity of the crude extracts greatly influence the efficacy. Batch-to-batch variations are high due to variable plantations conditions. Animal models of infection are limited
	Probiotics	Direct and indirect antiviral activity Immunoadjuvant effect Dual use as a vaccine-vector and immunomodulator	Efficacy against AIV particularly HPAIV is still questionable
**Molecular approaches**	**Avian Cytokines**	Not affected by antigenic changes Broad spectrum antiviral activities	Instability High production costs No mass production Field application limitations
	**RNA interference**	Inhibition of any influenza subtype/serotype/variant High specificity to particular strain/subtype/variant Do not require intact immune system Use as a prophylactic and/or therapeutic	Specificity to the viral genome without interference with the host genome and non-specifically inhibition of cellular gene activity is critical. Delivery to the host, costs, mass production, storage and handling of the final products consider questionable aspects. Possibility for arise of mutants with the ability to evade the siRNA activity should not be fully guaranteed Quickly degraded *in-vivo* Induce a transient & short-term protection and multiple-dose is required *In-vivo* research studies still missing
	**Naturally resistant birds **(Myxovirus Mx resistant gene and other candidate genes)	Few breeds of chickens and ducks can survive challenge with HPAIV in nature	Results on the contribution of the Mx gene to AIV resistant are contradictory Resistant breeds are mostly low producer native breeds. Interrelation of disease-resistance and production should be weighed Studies have been conducted only on a limited number of native breeds in some countries
	**Transgenic birds**	Although all infected transgenic birds succumbed to the infection however the virus did not spread to the in-contact transgenic and non-transgenic cagemates	Replacement of backyard flocks Consumer preferences Food safety Regulatory approval Costs of production Mutations of AIV

## References

[1] Palese P., Shaw M.L., Knipe D.M., Howley P.M. (2007). Orthomyxoviridae: The viruses and their replication. Fields Virology.

[2] Swayne D.E. (2009). Avian influenza vaccines and therapies for poultry. Comp. Immunol. Microbiol. Infect. Dis..

[3] Tong S., Li Y., Rivailler P., Conrardy C., Castillo D.A., Chen L.M., Recuenco S., Ellison J.A., Davis C.T., York I.A. (2012). A distinct lineage of influenza A virus from bats. Proc. Natl. Acad. Sci. U. S. A..

[4] Lupiani B., Reddy S.M. (2009). The history of avian influenza. Comp. Immunol. Microbiol. Infect. Dis..

[5] Brown E.G. (2000). Influenza virus genetics. Biomed. Pharmacother..

[6] Ferguson N.M., Galvani A.P., Bush R.M. (2003). Ecological and immunological determinants of influenza evolution. Nature.

[7] Webster R.G., Bean W.J., Gorman O.T., Chambers T.M., Kawaoka Y. (1992). Evolution and ecology of influenza A viruses. Microbiol. Rev..

[8] Peiris J.S., de Jong M.D., Guan Y. (2007). Avian influenza virus (H5N1): A threat to human health. Clin. Microbiol. Rev..

[9] Mumford E., Bishop J., Hendrickx S., Embarek P.B., Perdue M. (2007). Avian influenza H5N1: Risks at the human-animal interface. Food Nutr. Bull..

[10] Chen J., Chen S.C., Stern P., Scott B.B., Lois C. (2008). Genetic strategy to prevent influenza virus infections in animals. J. Infect. Dis..

[11] Yee K.S., Carpenter T.E., Cardona C.J. (2009). Epidemiology of H5N1 avian influenza. Comp. Immunol. Microbiol. Infect. Dis..

[12] Capua I., Alexander D.J. (2006). The challenge of avian influenza to the veterinary community. Avian Pathol..

[13] Swayne D.E., Pavade G., Hamilton K., Vallat B., Miyagishima K. (2011). Assessment of national strategies for control of high-pathogenicity avian influenza and low-pathogenicity notifiable avian influenza in poultry, with emphasis on vaccines and vaccination. Rev. Sci. Tech..

[14] van den Berg T., Lambrecht B., Marche S., Steensels M., Van Borm S., Bublot M. (2008). Influenza vaccines and vaccination strategies in birds. Comp. Immunol. Microbiol. Infect. Dis..

[15] Suarez D.L., Schultz-Cherry S. (2000). Immunology of avian influenza virus: A review. Dev. Comp. Immunol..

[16] Suarez D.L. (2005). Overview of avian influenza DIVA test strategies. Biologicals.

[17] Savill N.J., St Rose S.G., Keeling M.J., Woolhouse M.E. (2006). Silent spread of H5N1 in vaccinated poultry. Nature.

[18] Capua I., Alexander D.J. (2008). Ecology, epidemiology and human health implications of avian influenza viruses: Why do we need to share genetic data?. Zoonoses Public Hlth..

[19] Hafez M.H., Arafa A., Abdelwhab E.M., Selim A., Khoulosy S.G., Hassan M.K., Aly M.M. (2010). Avian influenza H5N1 virus infections in vaccinated commercial and backyard poultry in Egypt. Poultry Sci..

[20] Lee C.W., Senne D.A., Suarez D.L. (2004). Effect of vaccine use in the evolution of Mexican lineage H5N2 avian influenza virus. J. Virol..

[21] Cattoli G., Fusaro A., Monne I., Coven F., Joannis T., El-Hamid H.S., Hussein A.A., Cornelius C., Amarin N.M., Mancin M. (2011). Evidence for differing evolutionary dynamics of A/H5N1 viruses among countries applying or not applying avian influenza vaccination in poultry. Vaccine.

[22] Cattoli G., Milani A., Temperton N., Zecchin B., Buratin A., Molesti E., Aly M.M., Arafa A., Capua I. (2011). Antigenic drift in H5N1 avian influenza virus in poultry is driven by mutations in major antigenic sites of the hemagglutinin molecule analogous to those for human influenza virus. J. Virol..

[23] Boni M.F. (2008). Vaccination and antigenic drift in influenza. Vaccine.

[24] Escorcia M., Vazquez L., Mendez S.T., Rodriguez-Ropon A., Lucio E., Nava G.M. (2008). Avian influenza: Genetic evolution under vaccination pressure. Virol. J..

[25] Abdelwhab E.M., Grund C., Aly M.M., Beer M., Harder T.C., Hafez H.M. (2011). Multiple dose vaccination with heterologous H5N2 vaccine: Immune response and protection against variant clade 2.2.1 highly pathogenic avian influenza H5N1 in broiler breeder chickens. Vaccine.

[26] Grund C., Abdelwhab el S.M., Arafa A.S., Ziller M., Hassan M.K., Aly M.M., Hafez H.M., Harder T.C., Beer M. (2011). Highly pathogenic avian influenza virus H5N1 from Egypt escapes vaccine-induced immunity but confers clinical protection against a heterologous clade 2.2.1 Egyptian isolate. Vaccine.

[27] Kilany W.H., Abdelwhab E.M., Arafa A.S., Selim A., Safwat M., Nawar A.A., Erfan A.M., Hassan M.K., Aly M.M., Hafez H.M. (2011). Protective efficacy of H5 inactivated vaccines in meat turkey poults after challenge with Egyptian variant highly pathogenic avian influenza H5N1 virus. Vet. Microbiol..

[28] Rauw F., Palya V., Van Borm S., Welby S., Tatar-Kis T., Gardin Y., Dorsey K.M., Aly M.M., Hassan M.K., Soliman M.A. (2011). Further evidence of antigenic drift and protective efficacy afforded by a recombinant HVT-H5 vaccine against challenge with two antigenically divergent Egyptian clade 2.2.1 HPAI H5N1 strains. Vaccine.

[29] Swayne D.E., Kapczynski D. (2008). Strategies and challenges for eliciting immunity against avian influenza virus in birds. Immunol. Rev..

[30] Maas R., Rosema S., van Zoelen D., Venema S. (2011). Maternal immunity against avian influenza H5N1 in chickens: Limited protection and interference with vaccine efficacy. Avian Pathol..

[31] Sarfati-Mizrahi D., Lozano-Dubernard B., Soto-Priante E., Castro-Peralta F., Flores-Castro R., Loza-Rubio E., Gay-Gutierrez M. (2010). Protective dose of a recombinant Newcastle disease LaSota-avian influenza virus H5 vaccine against H5N2 highly pathogenic avian influenza virus and velogenic viscerotropic Newcastle disease virus in broilers with high maternal antibody levels. Avian Dis..

[32] Abdelwhab E.M., Grund C., Aly M.M., Beer M., Harder T.C., Hafez H.M. (2012). Influence of maternal immunity on vaccine efficacy and susceptibility of one day old chicks against Egyptian highly pathogenic avian influenza H5N1. Vet. Microbiol..

[33] De Vriese J., Steensels M., Palya V., Gardin Y., Dorsey K.M., Lambrecht B., Van Borm S., van den Berg T. (2010). Passive protection afforded by maternally-derived antibodies in chickens and the antibodies' interference with the protection elicited by avian influenza-inactivated vaccines in progeny. Avian Dis..

[34] Kim J.K., Kayali G., Walker D., Forrest H.L., Ellebedy A.H., Griffin Y.S., Rubrum A., Bahgat M.M., Kutkat M.A., Ali M.A. (2010). Puzzling inefficiency of H5N1 influenza vaccines in Egyptian poultry. Proc. Natl. Acad. Sci. U. S. A..

[35] Oh S., Martelli P., Hock O.S., Luz S., Furley C., Chiek E.J., Wee L.C., Keun N.M. (2005). Field study on the use of inactivated H5N2 vaccine in avian species. Vet. Rec..

[36] Philippa J.D., Munster V.J., Bolhuis H., Bestebroer T.M., Schaftenaar W., Beyer W.E., Fouchier R.A., Kuiken T., Osterhaus A.D. (2005). Highly pathogenic avian influenza (H7N7): Vaccination of zoo birds and transmission to non-poultry species. Vaccine.

[37] Cagle C., To T.L., Nguyen T., Wasilenko J., Adams S.C., Cardona C.J., Spackman E., Suarez D.L., Pantin-Jackwood M.J. (2011). Pekin and Muscovy ducks respond differently to vaccination with a H5N1 highly pathogenic avian influenza (HPAI) commercial inactivated vaccine. Vaccine.

[38] Lecu A., De Langhe C., Petit T., Bernard F., Swam H. (2009). Serologic response and safety to vaccination against avian influenza using inactivated H5N2 vaccine in zoo birds. J. Zoo Wildl. Med..

[39] Kapczynski D.R., Swayne D.E. (2009). Influenza vaccines for avian species. Curr. Top. Microbiol. Immunol..

[40] Koch G., Steensels M., van den Berg T. (2009). Vaccination of birds other than chickens and turkeys against avian influenza. Rev. Sci. Tech..

[41] Tian G., Zhang S., Li Y., Bu Z., Liu P., Zhou J., Li C., Shi J., Yu K., Chen H. (2005). Protective efficacy in chickens, geese and ducks of an H5N1-inactivated vaccine developed by reverse genetics. Virology.

[42] Bertelsen M.F., Klausen J., Holm E., Grondahl C., Jorgensen P.H. (2007). Serological response to vaccination against avian influenza in zoo-birds using an inactivated H5N9 vaccine. Vaccine.

[43] Robinson J.H., Easterday B.C. (1979). Avian influenza virus infection of the immunosuppressed turkey. Am. J. Vet. Res..

[44] Hao Y.X., Yang J.M., He C., Liu Q., McAllister T.A. (2008). Reduced serologic response to avian influenza vaccine in specific-pathogen-free chicks inoculated with Cryptosporidium baileyi. Avian Dis..

[45] Sun S., Cui Z., Wang J., Wang Z. (2009). Protective efficacy of vaccination against highly pathogenic avian influenza is dramatically suppressed by early infection of chickens with reticuloendotheliosis virus. Avian Pathol..

[46] Hegazy A.M., Abdallah F.M., Abd-El Samie L.K., Nazim A.A. (2011). The relation between some immunosuppressive agents and widespread nature of highly pathogenic avian influenza (HPAI) post vaccination. Am. J. Sci..

[47] Tolba M.K., Eskarous J.K. (1959). Response of some strains of Newcastle disease and fowl-plague viruses to two quinones. Arch. Mikrobiol..

[48] Moses H.E., Brandly C.A., Jones E.E., Jungherr E.L. (1948). The Isolation and Identification of Fowl Plague Virus. Am. J. Vet. Res..

[49] Sugrue R.J., Tan B.H., Yeo D.S., Sutejo R. (2008). Antiviral drugs for the control of pandemic influenza virus. Ann. Acad. Med. Singapore.

[50] Kamps B.S., Hoffman C., Kamps B.S., Hoffman C., Preiser W. (2006). Drug profiles. Influenza Report 2006.

[51] Kato N., Eggers H.J. (1969). Inhibition of uncoating of fowl plague virus by l-adamantanamine hydrochloride. Virology.

[52] Lang G., Narayan O., Rouse B.T. (1970). Prevention of malignant avian influenza by 1-adamantanamine hydrochloride. Arch. Gesamte Virusforsch..

[53] Beard C.W., Brugh M., Webster R.G. (1987). Emergence of amantadine-resistant H5N2 avian influenza virus during a simulated layer flock treatment program. Avian Dis..

[54] Webster R.G., Kawaoka Y., Bean W.J. (1986). Vaccination as a strategy to reduce the emergence of amantadine- and rimantadine-resistant strains of A/Chick/Pennsylvania/83 (H5N2) influenza virus. J. Antimicrob. Chemother..

[55] Bean W.J., Threlkeld S.C., Webster R.G. (1989). Biologic potential of amantadine-resistant influenza-a virus in an avian model. J. Infect. Dis..

[56] Bean W.J., Webster R.G. (1988). Biological properties of amantadine-resistant influenza-virus mutants. Antivir. Res..

[57] Scholtissek C., Faulkner G.P. (1979). Amantadine-resistant and -sensitive influenza A strains and recombinants. J. Gen. Virol..

[58] Bright R.A., Shay D.K., Shu B., Cox N.J., Klimov A.I. (2006). Adamantane resistance among influenza A viruses isolated early during the 2005–2006 influenza season in the United States. JAMA.

[59] Bright R.A., Medina M.J., Xu X., Perez-Oronoz G., Wallis T.R., Davis X.M., Povinelli L., Cox N.J., Klimov A.I. (2005). Incidence of adamantane resistance among influenza A (H3N2) viruses isolated worldwide from 1994 to 2005: A cause for concern. Lancet.

[60] Ilyushina N.A., Govorkova E.A., Webster R.G. (2005). Detection of amantadine-resistant variants among avian influenza viruses isolated in North America and Asia. Virology.

[61] Cheung C.L., Rayner J.M., Smith G.J., Wang P., Naipospos T.S., Zhang J., Yuen K.Y., Webster R.G., Peiris J.S., Guan Y. (2006). Distribution of amantadine-resistant H5N1 avian influenza variants in Asia. J. Infect. Dis..

[62] He G., Qiao J., Dong C., He C., Zhao L., Tian Y. (2008). Amantadine-resistance among H5N1 avian influenza viruses isolated in Northern China. Antivir. Res..

[63] Lan Y., Zhang Y., Dong L., Wang D., Huang W., Xin L., Yang L., Zhao X., Li Z., Wang W. (2010). A comprehensive surveillance of adamantane resistance among human influenza A virus isolated from mainland China between 1956 and 2009. Antivir. Ther..

[64] Tosh C., Murugkar H.V., Nagarajan S., Tripathi S., Katare M., Jain R., Khandia R., Syed Z., Behera P., Patil S. (2011). Emergence of amantadine-resistant avian influenza H5N1 virus in India. Virus Gene..

[65] Hurt A.C., Selleck P., Komadina N., Shaw R., Brown L., Barr I.G. (2007). Susceptibility of highly pathogenic A(H5N1) avian influenza viruses to the neuraminidase inhibitors and adamantanes. Antivir. Res..

[66] Cyranoski D. (2005). China's chicken farmers under fire for antiviral abuse. Nature.

[67] Parry J. (2005). Use of antiviral drug in poultry is blamed for drug resistant strains of avian flu. BMJ.

[68] Sipress A. (2005). Bird flu drug rendered useless. The Washington Post.

[69] Huang Y., Hu B., Wen X., Cao S., Xu D., Zhang X., Khan M.I. (2009). Evolution analysis of the matrix (M) protein genes of 17 H9N2 chicken influenza viruses isolated in northern China during 1998–2008. Virus Gene..

[70] Wainright P.O., Perdue M.L., Brugh M., Beard C.W. (1991). Amantadine resistance among hemagglutinin subtype 5 strains of avian influenza virus. Avian Dis..

[71] WHO World Health Organisation: Use of antiviral drugs in poultry, a threat to their effectiveness for the treatment of human avian influenza. http://www.who.int/foodsafety/micro/avian_antiviral/en/print.html.

[72] CDC (2006). Centers for Disease Control and Prevention: High levels of adamantane resistance among influenza A (H3N2) viruses and interim guidelines for use of antiviral agents—United States, 2005–06 influenza season. MMWR Morb. Mortal. Wkly. Rep..

[73] Webster R.G., Kawaoka Y., Bean W.J., Beard C.W., Brugh M. (1985). Chemotherapy and vaccination: A possible strategy for the control of highly virulent influenza virus. J. Virol..

[74] Allen U.D., Aoki F.Y., Stiver H.G. (2006). The use of antiviral drugs for influenza: Recommended guidelines for practitioners. Can. J. Infect. Dis. Med. Microbiol..

[75] McNicholl I.R., McNicholl J.J. (2001). Neuraminidase inhibitors: Zanamivir and oseltamivir. Ann. Pharmacother..

[76] Dreitlein W.B., Maratos J., Brocavich J. (2001). Zanamivir and oseltamivir: Two new options for the treatment and prevention of influenza. Clin. Therapeut..

[77] Ward P., Small I., Smith J., Suter P., Dutkowski R. (2005). Oseltamivir (Tamiflu) and its potential for use in the event of an influenza pandemic. J. Antimicrob. Chemother..

[78] Leneva I.A., Roberts N., Govorkova E.A., Goloubeva O.G., Webster R.G. (2000). The neuraminidase inhibitor GS4104 (oseltamivir phosphate) is efficacious against A/Hong Kong/156/97 (H5N1) and A/Hong Kong/1074/99 (H9N2) influenza viruses. Antivir. Res..

[79] de Jong M.D., Tran T.T., Truong H.K., Vo M.H., Smith G.J., Nguyen V.C., Bach V.C., Phan T.Q., Do Q.H., Guan Y. (2005). Oseltamivir resistance during treatment of influenza A (H5N1) infection. New Engl. J. Med..

[80] McKimm-Breschkin J.L., Selleck P.W., Usman T.B., Johnson M.A. (2007). Reduced sensitivity of influenza A (H5N1) to oseltamivir. Emerg. Infect. Dis..

[81] Hill A.W., Guralnick R.P., Wilson M.J., Habib F., Janies D. (2009). Evolution of drug resistance in multiple distinct lineages of H5N1 avian influenza. Infect. Genet. Evol..

[82] Earhart K.C., Elsayed N.M., Saad M.D., Gubareva L.V., Nayel A., Deyde V.M., Abdelsattar A., Abdelghani A.S., Boynton B.R., Mansour M.M. (2009). Oseltamivir resistance mutation N294S in human influenza A(H5N1) virus in Egypt. J. Infect. Publ. Health.

[83] Smith J.R. (2010). Oseltamivir in human avian influenza infection. J. Antimicrob. Chemother..

[84] Kayali G., Webby R.J., Ducatez M.F., El Shesheny R.A., Kandeil A.M., Govorkova E.A., Mostafa A., Ali M.A. (2011). The epidemiological and molecular aspects of influenza H5N1 viruses at the human-animal interface in Egypt. PLoS One.

[85] Meijer A., van der Goot A.J., Koch G., van Bovenc M., Kimman T.G. (2004). Oseltamivir reduces transmission, morbidity, and mortality of highly pathogenic avian influenza in chickens. Int. Congr..

[86] Kaleta E.F., Blanco Pena K.M., Yilmaz A., Redmann T., Hofheinz S. (2007). Avian influenza A viruses in birds of the order Psittaciformes: Reports on virus isolations, transmission experiments and vaccinations and initial studies on innocuity and efficacy of oseltamivir *in ovo*. DTW.

[87] Lee D.H., Lee Y.N., Park J.K., Yuk S.S., Lee J.W., Kim J.I., Han J.S., Lee J.B., Park S.Y., Choi I.S. (2011). Antiviral efficacy of oseltamivir against avian influenza virus in avian species. Avian Dis..

[88] Yen H.L., Ilyushina N.A., Salomon R., Hoffmann E., Webster R.G., Govorkova E.A. (2007). Neuraminidase inhibitor-resistant recombinant A/Vietnam/1203/04 (H5N1) influenza viruses retain their replication efficiency and pathogenicity *in vitro* and *in vivo*. J. Virol..

[89] Orozovic G., Orozovic K., Lennerstrand J., Olsen B. (2011). Detection of resistance mutations to antivirals oseltamivir and zanamivir in avian influenza A viruses isolated from wild birds. PLoS One.

[90] Moscona A. (2009). Global transmission of oseltamivir-resistant influenza. New Engl. J. Med..

[91] McKimm-Breschkin J., Trivedi T., Hampson A., Hay A., Klimov A., Tashiro M., Hayden F., Zambon M. (2003). Neuraminidase sequence analysis and susceptibilities of influenza virus clinical isolates to zanamivir and oseltamivir. Antimicrob. Agents Chemother..

[92] Guralnik M., Rosenbloom R.A., Petteruti M.P., Lefante C. (2007). Limitations of current prophylaxis against influenza virus infection. Am. J. Therapeut..

[93] Kitazato K., Wang Y., Kobayashi N. (2007). Viral infectious disease and natural products with antiviral activity. Drug Discov. Ther..

[94] Wang X., Jia W., Zhao A. (2006). Anti-influenza agents from plants and traditional Chinese medicine. Phytother. Res..

[95] Guo R., Pittler M.H., Ernst E. (2007). Complementary medicine for treating or preventing influenza or influenza-like illness. Am. J. Med..

[96] Chen W., Lim C.E., Kang H.J., Liu J. (2011). Chinese herbal medicines for the treatment of type A H1N1 influenza: A systematic review of randomized controlled trials. PLoS One.

[97] Nakayama M., Suzuki K., Toda M., Okubo S., Hara Y., Shimamura T. (1993). Inhibition of the infectivity of influenza virus by tea polyphenols. Antivir. Res..

[98] Kurokawa M., Kumeda C.A., Yamamura J., Kamiyama T., Shiraki K. (1998). Antipyretic activity of cinnamyl derivatives and related compounds in influenza virus-infected mice. Eur. J. Pharmacol..

[99] Mantani N., Andoh T., Kawamata H., Terasawa K., Ochiai H. (1999). Inhibitory effect of Ephedrae herba, an oriental traditional medicine, on the growth of influenza A/PR/8 virus in MDCK cells. Antivir. Res..

[100] Mantani N., Imanishi N., Kawamata H., Terasawa K., Ochiai H. (2001). Inhibitory effect of (+)-catechin on the growth of influenza A/PR/8 virus in MDCK cells. Planta Med..

[101] Imanishi N., Tuji Y., Katada Y., Maruhashi M., Konosu S., Mantani N., Terasawa K., Ochiai H. (2002). Additional inhibitory effect of tea extract on the growth of influenza A and B viruses in MDCK cells. Microbiol. Immunol..

[102] Jung K., Ha Y., Ha S.K., Han D.U., Kim D.W., Moon W.K., Chae C. (2004). Antiviral effect of Saccharomyces cerevisiae beta-glucan to swine influenza virus by increased production of interferon-gamma and nitric oxide. J. Vet. Med. B.

[103] Mak N.K., Leung C.Y., Wei X.Y., Shen X.L., Wong R.N., Leung K.N., Fung M.C. (2004). Inhibition of RANTES expression by indirubin in influenza virus-infected human bronchial epithelial cells. Biochem. Pharmacol..

[104] Kubo T., Nishimura H. (2007). Antipyretic effect of Mao-to, a Japanese herbal medicine, for treatment of type A influenza infection in children. Phytomedicine.

[105] Miki K., Nagai T., Suzuki K., Tsujimura R., Koyama K., Kinoshita K., Furuhata K., Yamada H., Takahashi K. (2007). Anti-influenza virus activity of biflavonoids. Bioorg. Med. Chem. Lett..

[106] Nagai T., Miyaichi Y., Tomimori T., Suzuki Y., Yamada H. (1992). *In vivo *anti-influenza virus activity of plant flavonoids possessing inhibitory activity for influenza virus sialidase. Antivir. Res..

[107] Kernan M.R., Sendl A., Chen J.L., Jolad S.D., Blanc P., Murphy J.T., Stoddart C.A., Nanakorn W., Balick M.J., Rozhon E.J. (1997). Two new lignans with activity against influenza virus from the medicinal plant Rhinacanthus nasutus. J. Nat. Prod..

[108] Quan F.S., Compans R.W., Cho Y.K., Kang S.M. (2007). Ginseng and Salviae herbs play a role as immune activators and modulate immune responses during influenza virus infection. Vaccine.

[109] Deryabin P.G., Lvov D.K., Botikov A.G., Ivanov V., Kalinovsky T., Niedzwiecki A., Rath M. (2008). Effects of a nutrient mixture on infectious properties of the highly pathogenic strain of avian influenza virus A/H5N1. Biofactors.

[110] Geng L., Shaozhong P., Shaohua Y., Ziren S., Xiaoping L. (2009). Experimental study on the antivirus effect of Zhongsheng pills on influenza virus H5N1. World Sci. Tech..

[111] Pleschka S., Stein M., Schoop R., Hudson J.B. (2009). Anti-viral properties and mode of action of standardized Echinacea purpurea extract against highly pathogenic avian influenza virus (H5N1, H7N7) and swine-origin H1N1 (S-OIV). Virol. J..

[112] Shin W.J., Lee K.H., Park M.H., Seong B.L. (2010). Broad-spectrum antiviral effect of Agrimonia pilosa extract on influenza viruses. Microbiol. Immunol..

[113] Sundararajan A., Ganapathy R., Huan L., Dunlap J.R., Webby R.J., Kotwal G.J., Sangster M.Y. (2010). Influenza virus variation in susceptibility to inactivation by pomegranate polyphenols is determined by envelope glycoproteins. Antivir. Res..

[114] He W., Han H., Wang W., Gao B. (2011). Anti-influenza virus effect of aqueous extracts from dandelion. Virol. J..

[115] Garozzo A., Timpanaro R., Stivala A., Bisignano G., Castro A. (2011). Activity of Melaleuca alternifolia (tea tree) oil on Influenza virus A/PR/8: Study on the mechanism of action. Antivir. Res..

[116] Glatthaar-Saalmuller B., Rauchhaus U., Rode S., Haunschild J., Saalmuller A. (2011). Antiviral activity* in vitro *of two preparations of the herbal medicinal product Sinupret(R) against viruses causing respiratory infections. Phytomedicine.

[117] Zhang L., Cheng Y.X., Liu A.L., Wang H.D., Wang Y.L., Du G.H. (2010). Antioxidant, anti-inflammatory and anti-influenza properties of components from Chaenomeles speciosa. Molecules.

[118] Kwon H.J., Kim H.H., Yoon S.Y., Ryu Y.B., Chang J.S., Cho K.O., Rho M.C., Park S.J., Lee W.S. (2010). *In vitro* inhibitory activity of Alpinia katsumadai extracts against influenza virus infection and hemagglutination. Virol. J..

[119] Wu Y., Li J.Q., Kim Y.J., Wu J., Wang Q., Hao Y. (2011). *In vivo *and *in vitro *antiviral effects of berberine on influenza virus. Chin. J. Integr. Med..

[120] Shaukat T.M., Ashraf M., Omer M.O., Rasheed M.A., Muhammad K., Shaukat T.M., Younus M., Shahzad M.K. (2011). Comparative efficacy of various antiviral agents against avian influenza virus (Type H7N3/Pakistan/2003). Pakistan J. Zool..

[121] Mehrbod P., Ideris A., Omar A.R., Hair-Bejo M., Tan S.W., Kheiri M.T., Tabatabaian M. (2012). Attenuation of influenza virus infectivity with herbal-marine compound (HESA-A): An *in vitro* study in MDCK cells. Virol. J..

[122] Sriwilaijaroen N., Fukumoto S., Kumagai K., Hiramatsu H., Odagiri T., Tashiro M., Suzuki Y. (2012). Antiviral effects of Psidium guajava Linn. (guava) tea on the growth of clinical isolated H1N1 viruses: Its role in viral hemagglutination and neuraminidase inhibition. Antivir. Res..

[123] Zu M., Yang F., Zhou W., Liu A., Du G., Zheng L. (2012). *In vitro *anti-influenza virus and anti-inflammatory activities of theaflavin derivatives. Antivir. Res..

[124] Garozzo A., Timpanaro R., Bisignano B., Furneri P.M., Bisignano G., Castro A. (2009). *In vitro* antiviral activity of Melaleuca alternifolia essential oil. Lett. Appl. Microbiol..

[125] Hudson J.B. (2009). The use of herbal extracts in the control of influenza. J. Med. Plants Res..

[126] Krawitz C., Mraheil M.A., Stein M., Imirzalioglu C., Domann E., Pleschka S., Hain T. (2011). Inhibitory activity of a standardized elderberry liquid extract against clinically-relevant human respiratory bacterial pathogens and influenza A and B viruses. BMC Compl. Alternative Med..

[127] Sood R., Swarup D., Bhatia S., Kulkarni D.D., Dey S., Saini M., Dubey S.C.  (2012). Antiviral activity of crude extracts of Eugenia jambolana Lam. against highly pathogenic avian influenza (H5N1) virus. Indian J. Exp. Biol..

[128] Gangopadhyay A., Ganguli S., Datta A. (2011). Inhibiting H5N1 hemagglutinin with samll molecule ligands. Int. J. Bioinformatics Res..

[129] Shang R.-F., Liang J.-P., Na Z.-Y., Yang H.-J., Lu Y., Hua L.-Y., Guo W.-Z., Cui Y., Wang L. (2010). *In vivo *inhibition of NAS preparation on H9N2 subtype AIV. Virol. Sin..

[130] Barbour E.K., Saadé M.F., Abdel Nour A.M., Kayali G., Kidess S., Bou Ghannam R., Harakeh S., Shaib H. (2011). Evaluation of essential oils in the treatment of broilers co-infected with multiple respiratory etiologic agents. Int. J. Appl. Res. Vet. Med..

[131] Barbour E.K., El-Hakim R.G., Kaadi M.S., Shaib H.A., Gerges D.D., Nehme P.A. (2006). Evaluation of the histopathology of the respiratory system in essential oil-treated broilers following a challenge with Mycoplasma gallisepticum and/or H9N2 influenza virus. Int. J. Appl. Res. Vet. Med..

[132] Lee H.J., Lee Y.N., Youn H.N., Lee D.H., Kwak J.H., Seong B.L., Lee J.B., Park S.Y., Choi I.S., Song C.S. (2012). Anti-influenza virus activity of green tea by-products* in vitro* and efficacy against influenza virus infection in chickens. Poultry Sci..

[133] Song J.M., Lee K.H., Seong B.L. (2005). Antiviral effect of catechins in green tea on influenza virus. Antivir. Res..

[134] Liu Z., Guo Z., Wang G., Zhang D., He H., Li G., Liu Y., Higgins D., Walsh A., Shanahan-Prendergast L. (2009). Evaluation of the efficacy and safety of a statin/caffeine combination against H5N1, H3N2 and H1N1 virus infection in BALB/c mice. Eur. J. Pharmaceut. Sci..

[135] Zhai L., Li Y., Wang W., Hu S. (2011). Enhancement of humoral immune responses to inactivated Newcastle disease and avian influenza vaccines by oral administration of ginseng stem-and-leaf saponins in chickens. Poultry Sci..

[136] Jiang W., Liu Y., Zheng H., Zheng Y., Xu H., Lu H. (2012). Immune regulation of avian influenza vaccine in hens using *Hypericum perforatum* L. methanol extraction. Plant Omics.

[137] Landy N., Ghalamkari G.H., Toghyani M. (2012). Evaluation of St John's Wort (Hypericum perforatum L.) as an antibiotic growth promoter substitution on performance, carcass characteristics, some of the immune responses, and serum biochemical parameters of broiler chicks. J. Med. Plants Res..

[138] Rajput Z.I., Xiao C.W., Hu S.H., Arijo A.G., Soomro N.M. (2007). Improvement of the efficacy of influenza vaccination (H5N1) in chicken by using extract of Cochinchina momordica seed (ECMS). J. Zhejiang Univ. Sci. B..

[139] Liu F.X., Sun S., Cui Z.Z. (2010). Analysis of immunological enhancement of immunosuppressed chickens by Chinese herbal extracts. J. Ethnopharmacol..

[140] Jafari R.A., Ghorbanpoor M., Hoshmand Diarjan S. (2009). Study on immunomodulatory activity of dietary garlic in chickens vaccinated against avian influenza virus (subtype H9N2). Int. J. Poultry Sci..

[141] Kurokawa M., Watanabe W., Shimizu T., Sawamura R., Shiraki K. (2010). Modulation of cytokine production by 7-hydroxycoumarin *in vitro* and its efficacy against influenza infection in mice. Antivir. Res..

[142] Fusco D., Liu X.Y., Savage C., Taur Y., Xiao W.L., Kennelly E., Yuan J.D., Cassileth B., Salvatore M., Papanicolaou G.A. (2010). Echinacea purpurea aerial extract alters course of influenza infection in mice. Vaccine.

[143] Hori T., Kiyoshima J., Shida K., Yasui H. (2001). Effect of intranasal administration of Lactobacillus casei Shirota on influenza virus infection of upper respiratory tract in mice. Clin. Diagn. Lab. Immunol..

[144] Yasui H., Kiyoshima J., Hori T. (2004). Reduction of influenza virus titer and protection against influenza virus infection in infant mice fed Lactobacillus casei Shirota. Clin. Diagn. Lab. Immunol..

[145] Olivares M., Diaz-Ropero M.P., Sierra S., Lara-Villoslada F., Fonolla J., Navas M., Rodriguez J.M., Xaus J. (2007). Oral intake of Lactobacillus fermentum CECT5716 enhances the effects of influenza vaccination. Nutrition.

[146] Boge T., Remigy M., Vaudaine S., Tanguy J., Bourdet-Sicard R., van der Werf S. (2009). A probiotic fermented dairy drink improves antibody response to influenza vaccination in the elderly in two randomised controlled trials. Vaccine.

[147] Harata G., He F., Hiruta N., Kawase M., Kubota A., Hiramatsu M., Yausi H. (2010). Intranasal administration of Lactobacillus rhamnosus GG protects mice from H1N1 influenza virus infection by regulating respiratory immune responses. Lett. Appl. Microbiol..

[148] Davidson L.E., Fiorino A.M., Snydman D.R., Hibberd P.L. (2011). Lactobacillus GG as an immune adjuvant for live-attenuated influenza vaccine in healthy adults: A randomized double-blind placebo-controlled trial. Eur. J. Clin. Nutr..

[149] Rizzardini G., Eskesen D., Calder P.C., Capetti A., Jespersen L., Clerici M. (2012). Evaluation of the immune benefits of two probiotic strains Bifidobacterium animalis ssp. lactis, BB-12(R) and Lactobacillus paracasei ssp. paracasei, L. casei 431(R) in an influenza vaccination model: A randomised, double-blind, placebo-controlled study. Br. J. Nutr..

[150] Iwabuchi N., Xiao J.Z., Yaeshima T., Iwatsuki K. (2011). Oral administration of Bifidobacterium longum ameliorates influenza virus infection in mice. Biol. Pharmaceut. Bull..

[151] Kawase M., He F., Kubota A., Yoda K., Miyazawa K., Hiramatsu M. (2012). Heat-killed Lactobacillus gasseri TMC0356 protects mice against influenza virus infection by stimulating gut and respiratory immune responses. FEMS Immunol. Med. Microbiol..

[152] Takeda S., Takeshita M., Kikuchi Y., Dashnyam B., Kawahara S., Yoshida H., Watanabe W., Muguruma M., Kurokawa M. (2011). Efficacy of oral administration of heat-killed probiotics from Mongolian dairy products against influenza infection in mice: Alleviation of influenza infection by its immunomodulatory activity through intestinal immunity. Int. Immunopharm..

[153] Patterson J.A., Burkholder K.M. (2003). Application of prebiotics and probiotics in poultry production. Poultry Sci..

[154] Nava G.M., Bielke L.R., Callaway T.R., Castaneda M.P. (2005). Probiotic alternatives to reduce gastrointestinal infections: The poultry experience. Anim. Health Res. Rev..

[155] Lutful Kabir S.M. (2009). The role of probiotics in the poultry industry. Int. J. Mol. Sci..

[156] Chon H., Choi B., Jeong G., Mo I. (2008). Evaluation system for an experimental study of low-pathogenic avian influenza virus (H9N2) infection in specific pathogen free chickens using lactic acid bacteria, Lactobacillus plantarum KFCC11389P. Avian Pathol..

[157] Seo B.J., Rather I.A., Kumar V.J., Choi U.H., Moon M.R., Lim J.H., Park Y.H. (2012). Evaluation of Leuconostoc mesenteroides YML003 as a probiotic against low-pathogenic avian influenza (H9N2) virus in chickens. J. Appl. Microbiol..

[158] Ghafoor A., Naseem S., Younus M., Nazir J. (2005). Immunomodulatory effects of multistrain probiotics (Protexin™) on broiler chicken vaccinated against avian influenza virus (H9). Int. J. Poultry Sci..

[159] Lei H., Xu Y., Chen J., Wei X., Lam D.M. (2010). Immunoprotection against influenza H5N1 virus by oral administration of enteric-coated recombinant Lactococcus lactis mini-capsules. Virology.

[160] Wang Z., Yu Q., Gao J., Yang Q. (2012). Mucosal and systemic immune responses induced by recombinant Lactobacillus spp. expressing the hemagglutinin of the avian influenza virus H5N1. Clin. Vaccine Immunol..

[161] Novak R., Ester K., Savic V., Sekellick M.J., Marcus P.I., Lowenthal J.W., Vainio O., Ragland W.L. (2001). Immune status assessment by abundance of IFN-alpha and IFN-gamma mRNA in chicken blood. J. Interferon Cytokine Res..

[162] Sekellick M.J., Ferrandino A.F., Hopkins D.A., Marcus P.I. (1994). Chicken interferon gene: Cloning, expression, and analysis. J. Interferon Res..

[163] Lukacsi K., Molnar M., Siroki O., Rosztoczy I. (1985). Combined effects of amantadine and interferon on influenza virus replication in chicken and human embryo trachea organ culture. Acta Microbiol. Hung..

[164] Marcus P.I., Girshick T., van der Heide L., Sekellick M.J. (2007). Super-sentinel chickens and detection of low-pathogenicity influenza virus. Emerg. Infect. Dis..

[165] Song L., Zhao D.G., Wu Y.J., Li Y. (2008). Transient expression of chicken alpha interferon gene in lettuce. J. Zhejiang Univ. Sci. B..

[166] Meng S., Yang L., Xu C., Qin Z., Xu H., Wang Y., Sun L., Liu W. (2011). Recombinant chicken interferon-alpha inhibits H9N2 avian influenza virus replication *in vivo* by oral administration. J. Interferon Cytokine Res..

[167] Wei Q., Peng G.Q., Jin M.L., Zhu Y.D., Zhou H.B., Guo H.Y., Chen H.C. (2006). Cloning, prokaryotic expression of chicken interferon-alpha gene and study on antiviral effect of recombinant chicken interferon-alpha. Chin. J. Biotechnol..

[168] Reemers S.S., van Haarlem D.A., Groot Koerkamp M.J., Vervelde L. (2009). Differential gene-expression and host-response profiles against avian influenza virus within the chicken lung due to anatomy and airflow. J. Gen. Virol..

[169] Sekellick M.J., Carra S.A., Bowman A., Hopkins D.A., Marcus P.I. (2000). Transient resistance of influenza virus to interferon action attributed to random multiple packaging and activity of NS genes. J. Interferon Cytokine Res..

[170] Xia C., Liu J., Wu Z.G., Lin C.Y., Wang M. (2004). The interferon-alpha genes from three chicken lines and its effects on H9N2 influenza viruses. Anim. Biotechnol..

[171] Rahman M.M., Uyangaa E., Han Y.W., Kim S.B., Kim J.H., Choi J.Y., Eo S.K. (2012). Oral co-administration of live attenuated Salmonella enterica serovar Typhimurium expressing chicken interferon-alpha and interleukin-18 enhances the alleviation of clinical signs caused by respiratory infection with avian influenza virus H9N2. Vet. Microbiol..

[172] Rahman M.M., Uyangaa E., Han Y.W., Kim S.B., Kim J.H., Choi J.Y., Yoo D.J., Hong J.T., Han S.B., Kim B. (2011). Oral administration of live attenuated Salmonella enterica serovar Typhimurium expressing chicken interferon-alpha alleviates clinical signs caused by respiratory infection with avian influenza virus H9N2. Vet. Microbiol..

[173] Chen H.Y., Shang Y.H., Yao H.X., Cui B.A., Zhang H.Y., Wang Z.X., Wang Y.D., Chao A.J., Duan T.Y. (2011). Immune responses of chickens inoculated with a recombinant fowlpox vaccine coexpressing HA of H9N2 avain influenza virus and chicken IL-18. Antivir. Res..

[174] Mingxiao M., Ningyi J., Zhenguo W., Ruilin W., Dongliang F., Min Z., Gefen Y., Chang L., Leili J., Kuoshi J. (2006). Construction and immunogenicity of recombinant fowlpox vaccines coexpressing HA of AIV H5N1 and chicken IL18. Vaccine.

[175] Chen H.Y., Cui B.A., Xia P.A., Li X.S., Hu G.Z., Yang M.F., Zhang H.Y., Wang X.B., Cao S.F., Zhang L.X. (2008). Cloning, *in vitro* expression and bioactivity of duck interleukin-18. Vet. Immunol. Immunopathol..

[176] Zhou J.Y., Wang J.Y., Chen J.G., Wu J.X., Gong H., Teng Q.Y., Guo J.Q., Shen H.G. (2005). Cloning, *in vitro *expression and bioactivity of duck interleukin-2. Mol. Immunol..

[177] Zhou J.Y., Chen J.G., Wang J.Y., Wu J.X., Gong H. (2005). cDNA cloning and functional analysis of goose interleukin-2. Cytokine.

[178] Wong J.P., Christopher M.E., Viswanathan S., Dai X., Salazar A.M., Sun L.Q., Wang M. (2009). Antiviral role of toll-like receptor-3 agonists against seasonal and avian influenza viruses. Curr. Pharmaceut. Des..

[179] Wong J.P., Christopher M.E., Viswanathan S., Karpoff N., Dai X., Das D., Sun L.Q., Wang M., Salazar A.M. (2009). Activation of toll-like receptor signaling pathway for protection against influenza virus infection. Vaccine.

[180] Stewart C.R., Bagnaud-Baule A., Karpala A.J., Lowther S., Mohr P.G., Wise T.G., Lowenthal J.W., Bean A.G. (2012). Toll-like receptor 7 ligands inhibit influenza A infection in chickens. J. Interferon Cytokine Res..

[181] Jenkins K.A., Lowenthal J.W., Kimpton W., Bean A.G. (2009). The *in vitro* and in ovo responses of chickens to TLR9 subfamily ligands. Dev. Comp. Immunol..

[182] Stram Y., Kuzntzova L. (2006). Inhibition of viruses by RNA interference. Virus Gene..

[183] Ge Q., Filip L., Bai A., Nguyen T., Eisen H.N., Chen J. (2004). Inhibition of influenza virus production in virus-infected mice by RNA interference. Proc. Natl. Acad. Sci. U. S. A..

[184] Ge Q., McManus M.T., Nguyen T., Shen C.H., Sharp P.A., Eisen H.N., Chen J. (2003). RNA interference of influenza virus production by directly targeting mRNA for degradation and indirectly inhibiting all viral RNA transcription. Proc. Natl. Acad. Sci. U. S. A..

[185] Sui H.Y., Zhao G.Y., Huang J.D., Jin D.Y., Yuen K.Y., Zheng B.J. (2009). Small interfering RNA targeting m2 gene induces effective and long term inhibition of influenza A virus replication. PLoS One.

[186] Zhou K., He H., Wu Y., Duan M. (2008). RNA interference of avian influenza virus H5N1 by inhibiting viral mRNA with siRNA expression plasmids. J. Biotechnol..

[187] Hui E.K., Yap E.M., An D.S., Chen I.S., Nayak D.P. (2004). Inhibition of influenza virus matrix (M1) protein expression and virus replication by U6 promoter-driven and lentivirus-mediated delivery of siRNA. J. Gen. Virol..

[188] Tompkins S.M., Lo C.Y., Tumpey T.M., Epstein S.L. (2004). Protection against lethal influenza virus challenge by RNA interference *in vivo*. Proc. Natl. Acad. Sci. U. S. A..

[189] Zhang W., Wang C.Y., Yang S.T., Qin C., Hu J.L., Xia X.Z. (2009). Inhibition of highly pathogenic avian influenza virus H5N1 replication by the small interfering RNA targeting polymerase A gene. Biochem. Biophys. Res. Comm..

[190] Zhou H., Jin M., Yu Z., Xu X., Peng Y., Wu H., Liu J., Liu H., Cao S., Chen H. (2007). Effective small interfering RNAs targeting matrix and nucleocapsid protein gene inhibit influenza A virus replication in cells and mice. Antivir. Res..

[191] Li Y.C., Kong L.H., Cheng B.Z., Li K.S. (2005). Construction of influenza virus siRNA expression vectors and their inhibitory effects on multiplication of influenza virus. Avian Dis..

[192] Abrahamyan A., Nagy E., Golovan S.P. (2009). Human H1 promoter expressed short hairpin RNAs (shRNAs) suppress avian influenza virus replication in chicken CH-SAH and canine MDCK cells. Antivir. Res..

[193] Bennink J.R., Palmore T.N. (2004). The promise of siRNAs for the treatment of influenza. Trends Mol. Med..

[194] Suzuki H., Saitoh H., Suzuki T., Takaku H. (2009). Inhibition of influenza virus by baculovirus-mediated shRNA. Nucleic Acids Symp. Ser. (Oxf).

[195] Ge Q., Eisen H.N., Chen J. (2004). Use of siRNAs to prevent and treat influenza virus infection. Virus Res..

[196] McSwiggen J.A., Seth S. (2008). A potential treatment for pandemic influenza using siRNAs targeting conserved regions of influenza A. Expet Opin. Biol. Ther..

[197] Elbashir S.M., Harborth J., Lendeckel W., Yalcin A., Weber K., Tuschl T. (2001). Duplexes of 21-nucleotide RNAs mediate RNA interference in cultured mammalian cells. Nature.

[198] Wadhwa R., Kaul S.C., Miyagishi M., Taira K. (2004). Know-how of RNA interference and its applications in research and therapy. Mutat. Res..

[199] Aigner A. (2006). Gene silencing through RNA interference (RNAi) *in vivo*: Strategies based on the direct application of siRNAs. J. Biotechnol..

[200] Morris K.V., Rossi J.J. (2006). Lentivirus-mediated RNA interference therapy for human immunodeficiency virus type 1 infection. Hum. Gene Ther..

[201] Thomas M., Ge Q., Lu J.J., Klibanov A.M., Chen J. (2005). Polycation-mediated delivery of siRNAs for prophylaxis and treatment of influenza virus infection. Expet Opin. Biol. Ther..

[202] O'Neill G. (2007). Australia tackles bird flu using RNAi. Nat. Biotechnol..

[203] Horby P., Nguyen N.Y., Dunstan S.J., Baillie J.K. (2012). The role of host genetics in susceptibility to influenza: A systematic review. PLoS One.

[204] Zekarias B., Ter Huurne A.A., Landman W.J., Rebel J.M., Pol J.M., Gruys E. (2002). Immunological basis of differences in disease resistance in the chicken. Vet. Res..

[205] Sironi L., Williams J.L., Moreno-Martin A.M., Ramelli P., Stella A., Jianlin H., Weigend S., Lombardi G., Cordioli P., Mariani P. (2008). Susceptibility of different chicken lines to H7N1 highly pathogenic avian influenza virus and the role of Mx gene polymorphism coding amino acid position 631. Virology.

[206] Swayne D.E., Radin M.J., Hoepf T.M., Slemons R.D. (1994). Acute renal failure as the cause of death in chickens following intravenous inoculation with avian influenza virus A/chicken/Alabama/7395/75 (H4N8). Avian Dis..

[207] Thomas C., Manin T.B., Andriyasov A.V., Swayne D.E. (2008). Limited susceptibility and lack of systemic infection by an H3N2 swine influenza virus in intranasally inoculated chickens. Avian Dis..

[208] Gharaibeh S. (2008). Pathogenicity of an avian influenza virus serotype H9N2 in chickens. Avian Dis..

[209] Keawcharoen J., van Riel D., van Amerongen G., Bestebroer T., Beyer W.E., van Lavieren R., Osterhaus A.D., Fouchier R.A., Kuiken T. (2008). Wild ducks as long-distance vectors of highly pathogenic avian influenza virus (H5N1). Emerg. Infect. Dis..

[210] Munster V.J., Baas C., Lexmond P., Waldenstrom J., Wallensten A., Fransson T., Rimmelzwaan G.F., Beyer W.E., Schutten M., Olsen B. (2007). Spatial, temporal, and species variation in prevalence of influenza A viruses in wild migratory birds. PLoS Pathog..

[211] Brown J.D., Stallknecht D.E., Beck J.R., Suarez D.L., Swayne D.E. (2006). Susceptibility of North American ducks and gulls to H5N1 highly pathogenic avian influenza viruses. Emerg. Infect. Dis..

[212] Ruff M. (1983). Interferon-mediated development of influenza virus resistance in hybrids between Mx gene-bearing and control mouse embryo fibroblasts. J. Gen. Virol..

[213] Staeheli P., Haller O., Boll W., Lindenmann J., Weissmann C. (1986). Mx protein: Constitutive expression in 3T3 cells transformed with cloned Mx cDNA confers selective resistance to influenza virus. Cell.

[214] Chang K.C., Goldspink G., Lida J. (1990). Studies in the *in vivo* expression of the influenza resistance gene Mx by in-situ hybridisation. Arch. Virol..

[215] Meier E., Kunz G., Haller O., Arnheiter H. (1990). Activity of rat Mx proteins against a rhabdovirus. J. Virol..

[216] Salomon R., Staeheli P., Kochs G., Yen H.L., Franks J., Rehg J.E., Webster R.G., Hoffmann E. (2007). Mx1 gene protects mice against the highly lethal human H5N1 influenza virus. Cell Cycle.

[217] Haller O., Staeheli P., Kochs G. (2009). Protective role of interferon-induced Mx GTPases against influenza viruses. Rev. Sci. Tech..

[218] Pavlovic J., Zurcher T., Haller O., Staeheli P. (1990). Resistance to influenza virus and vesicular stomatitis virus conferred by expression of human MxA protein. J. Virol..

[219] Ko J.H., Jin H.K., Asano A., Takada A., Ninomiya A., Kida H., Hokiyama H., Ohara M., Tsuzuki M., Nishibori M. (2002). Polymorphisms and the differential antiviral activity of the chicken Mx gene. Genome Res..

[220] Li X.Y., Qu L.J., Yao J.F., Yang N. (2006). Skewed allele frequencies of an Mx gene mutation with potential resistance to avian influenza virus in different chicken populations. Poultry Sci..

[221] Li X.Y., Qu L.J., Hou Z.C., Yao J.F., Xu G.Y., Yang N. (2007). Genomic structure and diversity of the chicken Mx gene. Poultry Sci..

[222] Watanabe T. (2007). Polymorphisms of the chicken antiviral MX gene. Cytogenet. Genome Res..

[223] Sartika T., Sulandari S., Zein M.S. (2011). Selection of Mx gene genotype as genetic marker for Avian Influenza resistance in Indonesian native chicken. BMC Proc..

[224] Dillon D., Runstadler J.  (2010). Mx gene diversity and influenza association among five wild dabbling duck species (Anas spp.) in Alaska. Infect. Genet. Evol..

[225] Berlin S., Qu L., Li X., Yang N., Ellegren H. (2008). Positive diversifying selection in avian Mx genes. Immunogenetics.

[226] Yin C.G., Zhang C.S., Zhang A.M., Qin H.W., Wang X.Q., Du L.X., Zhao G.P. (2010). Expression analyses and antiviral properties of the Beijing-You and White Leghorn myxovirus resistance gene with different amino acids at position 631. Poultry Sci..

[227] Seyama T., Ko J.H., Ohe M., Sasaoka N., Okada A., Gomi H., Yoneda A., Ueda J., Nishibori M., Okamoto S. (2006). Population research of genetic polymorphism at amino acid position 631 in chicken Mx protein with differential antiviral activity. Biochem. Genet..

[228] Balkissoon D., Staines K., McCauley J., Wood J., Young J., Kaufman J., Butter C. (2007). Low frequency of the Mx allele for viral resistance predates recent intensive selection in domestic chickens. Immunogenetics.

[229] Bazzigher L., Schwarz A., Staeheli P. (1993). No enhanced influenza virus resistance of murine and avian cells expressing cloned duck Mx protein. Virology.

[230] Schumacher B., Bernasconi D., Schultz U., Staeheli P. (1994). The chicken Mx promoter contains an ISRE motif and confers interferon inducibility to a reporter gene in chick and monkey cells. Virology.

[231] Ewald S.J., Kapczynski D.R., Livant E.J., Suarez D.L., Ralph J., McLeod S., Miller C. (2011). Association of Mx1 Asn631 variant alleles with reductions in morbidity, early mortality, viral shedding, and cytokine responses in chickens infected with a highly pathogenic avian influenza virus. Immunogenetics.

[232] Benfield C.T., Lyall J.W., Kochs G., Tiley L.S. (2008). Asparagine 631 variants of the chicken Mx protein do not inhibit influenza virus replication in primary chicken embryo fibroblasts or *in vitro* surrogate assays. J. Virol..

[233] Benfield C.T., Lyall J.W., Tiley L.S. (2010). The cytoplasmic location of chicken mx is not the determining factor for its lack of antiviral activity. PLoS One.

[234] Schusser B., Reuter A., von der Malsburg A., Penski N., Weigend S., Kaspers B., Staeheli P., Hartle S. (2011). Mx is dispensable for interferon-mediated resistance of chicken cells against influenza A virus. J. Virol..

[235] Bernasconi D., Schultz U., Staeheli P. (1995). The interferon-induced Mx protein of chickens lacks antiviral activity. J. Interferon Cytokine Res..

[236] Sironi L., Williams J.L., Stella A., Minozzi G., Moreno A., Ramelli P., Han J., Weigend S., Wan J., Lombardi G. (2011). Genomic study of the response of chicken to highly pathogenic avian influenza virus. BMC Proc..

[237] Qu L.J., Li X.Y., Xu G.Y., Ning Z.H., Yang N. (2009). Lower antibody response in chickens homozygous for the Mx resistant allele to avian influenza. Asian-Aust. J. Anim. Sci..

[238] Barber M.R., Aldridge J.R., Webster R.G., Magor K.E. (2010). Association of RIG-I with innate immunity of ducks to influenza. Proc. Natl. Acad. Sci. U. S. A..

[239] Karpala A.J., Stewart C., McKay J., Lowenthal J.W., Bean A.G. (2011). Characterization of chicken Mda5 activity: Regulation of IFN-beta in the absence of RIG-I functionality. J. Immunol..

[240] Liniger M., Summerfield A., Zimmer G., McCullough K.C., Ruggli N. (2012). Chicken cells sense influenza A virus infection through MDA5 and CARDIF signaling involving LGP2. J. Virol..

[241] Adams S.C., Xing Z., Li J., Cardona C.J. (2009). Immune-related gene expression in response to H11N9 low pathogenic avian influenza virus infection in chicken and Pekin duck peripheral blood mononuclear cells. Mol. Immunol..

[242] Sarmento L., Afonso C.L., Estevez C., Wasilenko J., Pantin-Jackwood M. (2008). Differential host gene expression in cells infected with highly pathogenic H5N1 avian influenza viruses. Vet. Immunol. Immunopathol..

[243] Liang Q.L., Luo J., Zhou K., Dong J.X., He H.X. (2011). Immune-related gene expression in response to H5N1 avian influenza virus infection in chicken and duck embryonic fibroblasts. Mol. Immunol..

[244] Kuchipudi S.V., Dunham S.P., Nelli R., White G.A., Coward V.J., Slomka M.J., Brown I.H., Chang K.C. (2012). Rapid death of duck cells infected with influenza: A potential mechanism for host resistance to H5N1. Immunol. Cell Biol..

[245] Xu C., Meng S., Liu X., Sun L., Liu W. (2010). Chicken cyclophilin A is an inhibitory factor to influenza virus replication. Virol. J..

[246] Hsiang T.Y., Zhao C., Krug R.M. (2009). Interferon-induced ISG15 conjugation inhibits influenza A virus gene expression and replication in human cells. J. Virol..

[247] Wang X., Hinson E.R., Cresswell P. (2007). The interferon-inducible protein viperin inhibits influenza virus release by perturbing lipid rafts. Cell Host Microbe.

[248] Watanabe K., Fuse T., Asano I., Tsukahara F., Maru Y., Nagata K., Kitazato K., Kobayashi N. (2006). Identification of Hsc70 as an influenza virus matrix protein (M1) binding factor involved in the virus life cycle. FEBS Lett..

[249] Honda A., Okamoto T., Ishihama A. (2007). Host factor Ebp1: Selective inhibitor of influenza virus transcriptase. Gene. Cell..

[250] Scott B.B., Lois C. (2005). Generation of tissue-specific transgenic birds with lentiviral vectors. Proc. Natl. Acad. Sci. U. S. A..

[251] Harvey A.J., Speksnijder G., Baugh L.R., Morris J.A., Ivarie R. (2002). Consistent production of transgenic chickens using replication-deficient retroviral vectors and high-throughput screening procedures. Poultry Sci..

[252] Lyall J., Irvine R.M., Sherman A., McKinley T.J., Nunez A., Purdie A., Outtrim L., Brown I.H., Rolleston-Smith G., Sang H. (2011). Suppression of avian influenza transmission in genetically modified chickens. Science.

[253] Enserink M. (2011). Avian influenza. Transgenic chickens could thwart bird flu, curb pandemic risk. Science.

[254] Boon A.C., deBeauchamp J., Hollmann A., Luke J., Kotb M., Rowe S., Finkelstein D., Neale G., Lu L., Williams R.W. (2009). Host genetic variation affects resistance to infection with a highly pathogenic H5N1 influenza A virus in mice. J. Virol..

